# Synthesis, Anti-Inflammatory Activities, and Molecular Docking Study of Novel Pyxinol Derivatives as Inhibitors of NF-κB Activation

**DOI:** 10.3390/molecules29081711

**Published:** 2024-04-10

**Authors:** Shuai Tan, Zongji Zou, Xuwen Luan, Cheng Chen, Shuang Li, Zhen Zhang, Mengran Quan, Xiang Li, Wei Zhu, Gangqiang Yang

**Affiliations:** School of Pharmacy, Collaborative Innovation Center of Advanced Drug Delivery System and Biotech Drugs in Universities of Shandong, Key Laboratory of Molecular Pharmacology and Drug Evaluation (Yantai University), Ministry of Education, Yantai University, Yantai 264005, China; tanshuai317533862@163.com (S.T.); 15666432573@163.com (Z.Z.); arrebol98@163.com (X.L.); cc252413469@163.com (C.C.); shuangli202301@163.com (S.L.); dania163com@icloud.com (Z.Z.); 19558912690@163.com (M.Q.); 15753861978@163.com (X.L.)

**Keywords:** pyxinol derivatives, anti-inflammatory activity, ginsenosides, synthesis, NF-κB, molecular docking, MtROS

## Abstract

Pyxinol, an active metabolite of ginsenosides in human hepatocytes, exhibits various pharmacological activities. Here, a series of C-3 modified pyxinol derivatives was designed and virtually screened by molecular docking with the key inflammation-related proteins of the nuclear factor kappa B (NF-κB) pathway. Some of the novel derivatives were synthesized to assess their effects in inhibiting the production of nitric oxide (NO) and mitochondrial reactive oxygen species (MtROS) in lipopolysaccharide-triggered RAW264.7 cells. Derivative **2c** exhibited the highest NO and MtROS inhibitory activities with low cytotoxicity. Furthermore, **2c** decreased the protein levels of interleukin 1β, tumor necrosis factor α, inducible nitric oxide synthase, and cyclooxygenase 2 and suppressed the activation of NF-κB signaling. Cellular thermal shift assays indicated that **2c** could directly bind with p65 and p50 in situ. Molecular docking revealed that **2c**’s binding to the p65–p50 heterodimer and p50 homodimer was close to their DNA binding sites. In summary, pyxinol derivatives possess potential for development as NF-κB inhibitors.

## 1. Introduction

Lipopolysaccharide (LPS)-triggered inflammation is an essential defense process against infection, and nuclear factor-kappa B (NF-κB) plays a key regulatory role in this process [1,2,3]. The gene expression of major proinflammatory cytokines, such as tumor necrosis factor-α (TNF-α) and interleukins (ILs), is mainly regulated by NF-κB in inflammation. Activated NF-κB, which is mainly present in the form of a p65–p50 heterodimer, transactivates major proinflammatory genes, including *interleukins* and *TNF-α* [4]. Conversely, the p50 homodimer, an atypical NF-κB species, lacks the transactivation domain and impedes the expression of NF-κB’s target gene by competing with the p65–p50 heterodimer for DNA binding [4,5]. Additionally, the prolonged production of proinflammatory cytokines causes local and systemic damage in dysregulated inflammation [2]. Hence, NF-κB is a key drug target in overcoming dysregulated inflammation.

**Figure 1:**
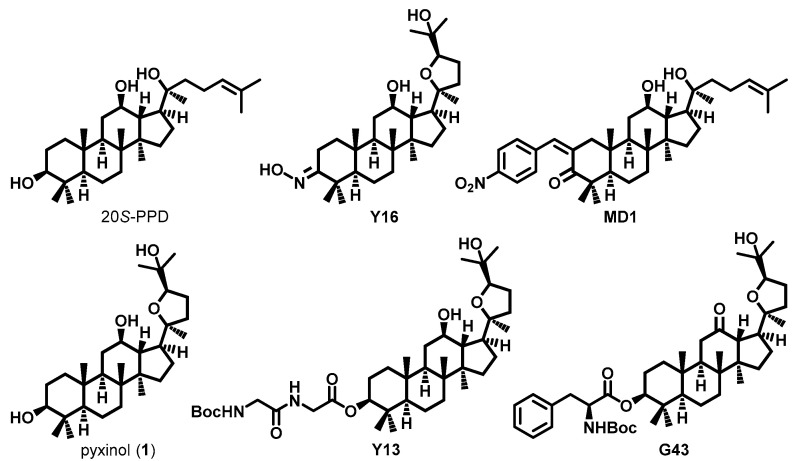
Structures of 20*S*-PPD, pyxinol, and its derivatives.

Ginsenosides are the main active ingredients of ginseng, a well-known edible herb, and they exhibit various pharmacological activities [6,7]. Their anti-inflammatory activity is remarkable owing to the structural similarity between aglycones and glucocorticoids (the most effective anti-inflammatory agents) [8,9]. 20*S*-Protopanoxadiol (20*S*-PPD), one of the main intestinal metabolites of ginsenosides, is an aglycone that can be developed as a potent anti-inflammatory agent [8,10]. Recently, pyxinol (Figure 1) has garnered increasing attention because it is a key metabolite of 20*S*-PPD in human hepatocytes and exhibits better oral bioavailability and a lower metabolism burden than 20*S*-PPD [11,12,13]. Although the core skeletons of pyxinol and 20*S*-PPD are almost the same, the C-20 position of pyxinol is a tetrahydrofuran ring, whereas that of 20*S*-PPD is a carbon chain. A series of pyxinol derivatives exhibiting various pharmacological activities, including cardioprotective, multidrug resistance-reversal, and antibiotic effects, has been developed [14,15,16,17,18,19,20,21,22,23,24,25,26,27]. Specifically, great attention has been paid to pyxinol derivatives with anti-inflammatory activity because their activity is superior to that of hydrocortisone sodium succinate (HSS), a glucocorticoid drug [28,29,30]. Derivative **Y16** suppresses inflammation via the NF-κB pathways [30]. Similar to 20*S*-PPD and the parental ginsenosides [31,32], **Y13** and **G43** exert anti-inflammatory activity by inhibiting the NF-κB and mitogen-activated protein kinase pathways [28,29]. The anti-inflammatory activity of **G43** is independent of the glucocorticoid receptor pathway [28]. All these pyxinol derivatives with potent anti-inflammatory activity are derived from the C-3 hydroxyl group of the core skeleton and have an *R*-configuration at C-24 [28,29,30]. **MD1**, a derivative of the A-ring of 20*S*-PPD, exhibits potent anti-inflammatory activity by primarily acting via the nucleotide-binding domain, the leucine-rich–containing family, the pyrin domain-containing-3 pathway [10]. Additionally, it is associated with the NF-κB pathway. However, the targets and the detailed molecular mechanisms of these derivatives for anti-inflammatory activity remain unclear. Consequently, guiding the rational design of efficient derivatives is challenging.

The abovementioned derivatives of pyxinol and 20*S*-PPD have been freely designed and synthesized. Moreover, their anti-inflammatory activity is more or less associated with the NF-κB pathway. Therefore, NF-κB can be a potential drug target of these derivatives for reducing inflammation. Here, we designed and synthesized novel pyxinol analogs targeting NF-κB and analyzed their molecular mechanisms by performing molecular docking. First, a series of pyxinol derivatives with a small molecular fragment modified at the C-3 hydroxyl was designed and linked with an ester bond to facilitate synthesis. Second, the p65–p50 heterodimer (PDB: 1VKX) [33] and the p50 homodimer (PDB: 1NFK) [34] were used as targets for the virtual screening of potential active derivatives by performing molecular docking. Some of the derivatives were synthesized to examine their suppressive effects on LPS-triggered inflammation in vitro. Finally, a cellular thermal shift assay (CETSA) was performed to verify interactions between NF-κB and the potent derivative, which is a label-free approach to efficiently evaluate protein–compound interactions in situ [35,36]. Their binding and molecular mechanisms in reducing inflammation were further analyzed by performing molecular docking.

## 2. Results and Discussion

### 2.1. Designing Pyxinol Derivatives and Virtual Screening

Substituted benzene ring groups widely exist in the anti-inflammatory drugs and compounds with potent anti-inflammatory activity. Compounds modified with heterocyclic molecule fragments, including pyrrole, pyrazole, oxazole, thiazole, furan, and indole, exhibit negligible cytotoxicity while developing anti-inflammatory agents [37]. Thus, these molecular fragments were used in the present study to construct a library of virtual C-3 hydroxyl esterification derivatives of pyxinol. Additionally, the library contained the reported pyxinol derivatives [10,28,29,30] with anti-inflammatory activity.

**Figure 2:**
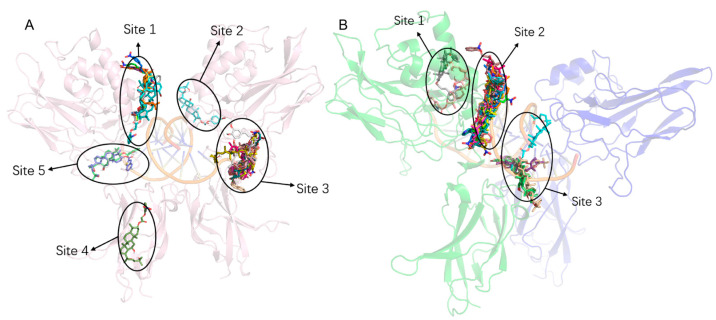
Predicted binding modes for selected pyxinol derivatives with (**A**) the p50 homodimer (PDB: 1NFK) and (**B**) the p65–p50 heterodimer (PDB: 1VKX).

**Scheme 1:**
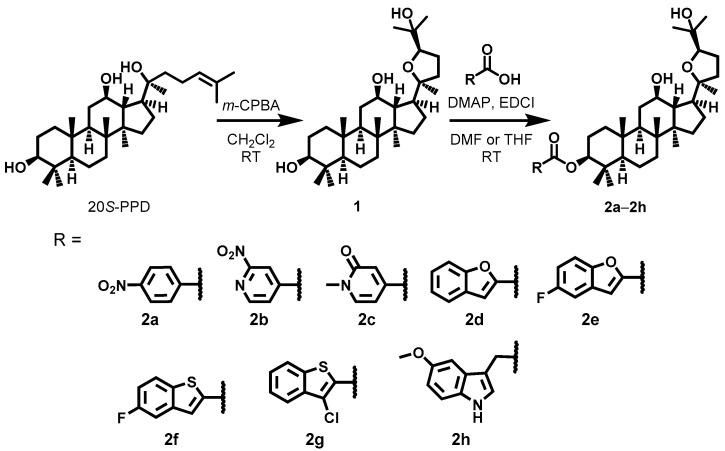
Synthesis of pyxinol derivatives.

A molecular docking analysis was performed using the p65–p50 heterodimer (PDB: 1VKX) and p50 homodimer (PDB: 1NFK) to identify potential active pyxinol derivatives targeting NF-κB. The highest estimated binding affinity between a pyxinol derivative and NF-κB was calculated using AutoDock 4.0 from 100 replicates per derivative. A pyxinol derivative bound to NF-κB with a minimum estimated Δ*G*_binding_ value less than −7.0 kcal/mol was selected. The binding sites of the majority of the selected derivatives were close to the DNA binding sites [33,34] of both the heterodimer and homodimer, suggesting that the derivatives may exhibit anti-inflammatory activities by interfering with the binding of activated NF-κB (p65–p50 heterodimer) to DNA (Figure 2). Additionally, this result indicated that our prediction was feasible. Novel derivatives with a compacted linker (Scheme 1) were synthesized for further verification. Their minimum estimated Δ*G*_binding_ values are presented in Table 1.

### 2.2. Synthesis

Pyxinol (**1**) was prepared using commercial 20*S*-PPD by one-step epoxidation and purified by recrystallization as described previously [16,38] (Scheme 1). The C-3-selective esterification of pyxinol (**1**) with the corresponding carboxylic acid was catalyzed by 4-dimethylaminopyridine (DMAP) in dried DMF or THF to synthesize the target derivatives (**2a**–**2h**). The structures of the final pyxinol derivatives were verified by ^1^H-nuclear magnetic resonance (NMR), ^13^C-NMR, and high-resolution mass spectroscopy (HRMS).

### 2.3. Inhibition of LPS-Triggered Nitric Oxide (NO) Release

**Figure 3:**
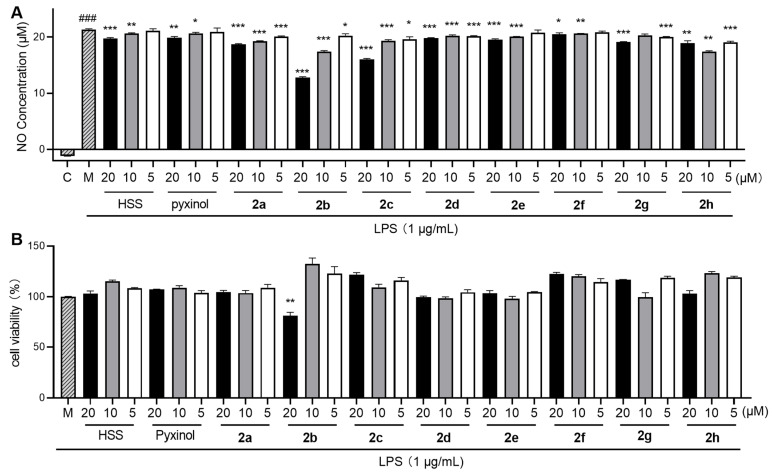
Inhibition of pyxinol derivatives on the LPS-triggered release of NO. (**A**) The effect of pyxinol derivatives on the release of NO and (**B**) cell survival in LPS-triggered RAW264.7 macrophages. The macrophages were seeded into a 96-well plate, pre-treated with pyxinol derivatives or HSS (5, 10, and 20 μM) for 2 h, and then treated with LPS (1 μg/mL) for 24 h. Nitrite concentrations and cell viability were then measured. Data are expressed as the means ± SD (n = 3): * *p* < 0.05, ** *p* < 0.01, *** *p* < 0.001 vs. the model (M) group. ### *p* < 0.001 vs. the control (C) group.

The release of NO is a typical inflammatory response elicited in various inflammatory diseases. It is a key indicator used to evaluate the anti-inflammatory activity of a compound [39,40]. Here, we evaluated the NO inhibition activities of all synthesized pyxinol derivatives in LPS-triggered RAW264.7 cells, and HSS was used as a positive control. The Griess assay results confirmed that LPS (1 μg/mL) largely triggered the release of NO (Figure 3A). Except for derivative **2f**, the remaining derivatives significantly inhibited the LPS-triggered release of NO (*p* < 0.05) and exhibited stronger inhibitory activity than did the parental pyxinol in a dose-dependent manner. Of these, the size of the modification moiety obviously affected the NO-inhibitory activity of the derivatives (**2a**–**2c** vs. **2d**–**2g**). The introduction of a linker reduced the effect of the large modification moiety on its NO-inhibitory activity (**2h** vs. **2d**–**2g**). Notably, derivatives **2b** and **2c** had the most potent suppression of the LPS-triggered release of NO, which was better than the ability of HSS.

The cytotoxicity of all synthesized pyxinol derivatives was evaluated in LPS-treated macrophages to confirm whether the non-specific inhibition of NO release was because of cytotoxicity-induced cell death. Except for **2b**, no other derivatives exhibited cytotoxicity at 20 μM (Figure 3B). Compared with the control, treatment with 20 μM of **2b** resulted in cell viabilities of ~80%, and when its concentration was reduced to 10 μM, the cells’ viability was higher than 100%. These findings suggest that pyxinol derivatives suppress the LPS-triggered release of NO owing to their anti-inflammatory activity.

### 2.4. Inhibition of LPS-Triggered Generation of Mitochondrial Reactive Oxygen Species (MtROS) 

**Figure 4:**
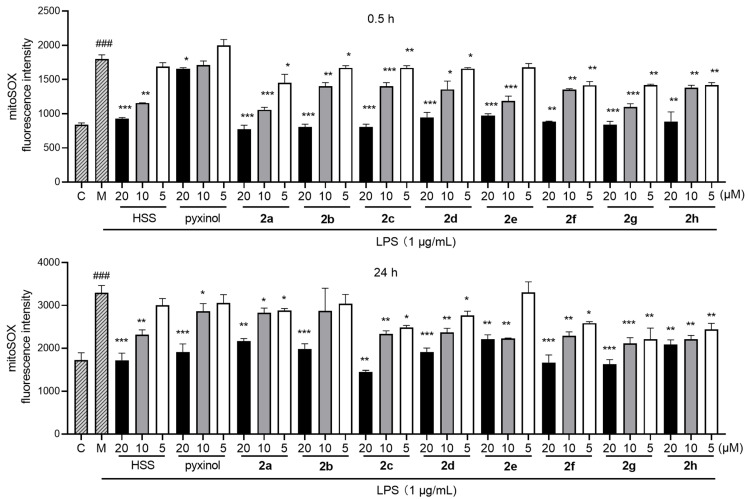
Inhibition of pyxinol derivatives on the LPS-triggered generation of MtROS in RAW264.7 macrophages. After pre-treatment with pyxinol derivatives or HSS (5, 10, and 20 μM) for 2 h, the macrophages were treated with LPS for another 0.5 or 24 h. The MtROS were determined by the mitoSOX assay. Data are expressed as the means ± SD (n = 3): * *p* < 0.05, ** *p* < 0.01, *** *p* < 0.001 vs. the model (M) group. ### *p* < 0.001 vs. the control (C) group.

Inflammation can change in the cell microenvironment by increasing the generation of ROS [41]. Moreover, ROS promotes inflammation, resulting in a vicious cycle [41]. In particular, LPS-triggered generation of MtROS is an alternative key inflammatory response that causes cell damage [42,43]. A mitoSOX assay was performed to evaluate the inhibitory effect of the derivatives on LPS-triggered generation of MtROS. LPS treatment markedly triggered the generation of MtROS in the macrophages, evidenced by an increase in the fluorescence intensity of mitoSOX during short-term (0.5 h) and long-term (24 h) treatment (Figure 4) in a dose-dependent manner. Most of the pyxinol derivatives had better inhibitory activity than pyxinol. Derivative **2c** showed the most potent ability to suppress the LPS-triggered generation of MtROS, which was better than that of HSS, especially during short-term treatment. Thus, **2c** displayed the highest NO- and MtROS-inhibitory activities with low cytotoxicity and was selected for further studies.

### 2.5. Inhibition of LPS-Triggered Cytokine Release

**Figure 5:**
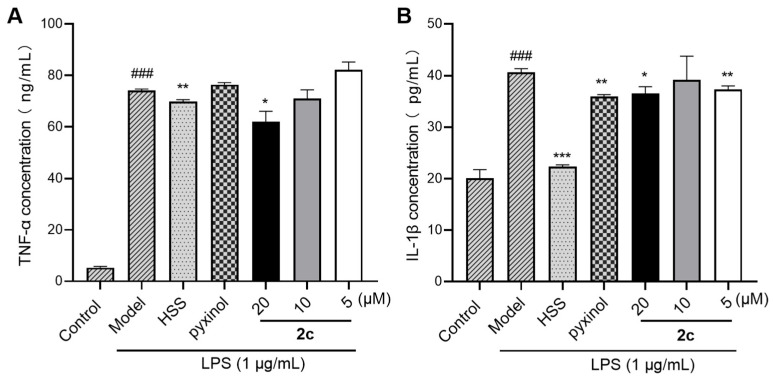
Effect of derivative **2c** on the LPS-triggered release of (**A**) TNF-α and (**B**) IL-1β in RAW264.7 macrophages. The macrophages were pre-treated with **2c** (5, 10 and 20 μM) or HSS (20 μM) for 2 h before 6 (for TNF-α) or 24 h (for IL-1β) of stimulation with LPS (1 μg/mL). The levels of TNF-α and IL-1β were analyzed using Elabscience ELISA kits. Data are expressed as the means ± SD (n = 3): ### *p* < 0.001 vs. the control group; * *p* < 0.05, ** *p* < 0.01, *** *p* < 0.001 vs. the model group.

TNF-α and IL-1β are key pro-inflammatory cytokines produced during inflammation that elicit further inflammatory responses, which are generated and mediated by the activation of NF-κB [44]. Next, we evaluated the ability of **2c** to inhibit the LPS-triggered release of TNF-α and IL-1β by performing an enzyme-linked immunosorbent assay (ELISA) to further confirm the anti-inflammatory activity of **2c**. Compared with the control, LPS (1 μg/mL) rapidly triggered the release of TNF-α and IL-1β in macrophages (Figure 5). Furthermore, treatment with **2c** significantly inhibited the LPS-triggered release of TNF-α and IL-1β (*p* < 0.05), showing stronger inhibitory activity on the release of TNF-α than that of HSS. These findings confirmed that **2c** exhibits potent anti-inflammatory activities.

### 2.6. Inhibition of LPS-Triggered Generation of Inducible Nitric Oxide Synthase (iNOS) and Cyclooxygenase-2 (COX-2) 

**Figure 6:**
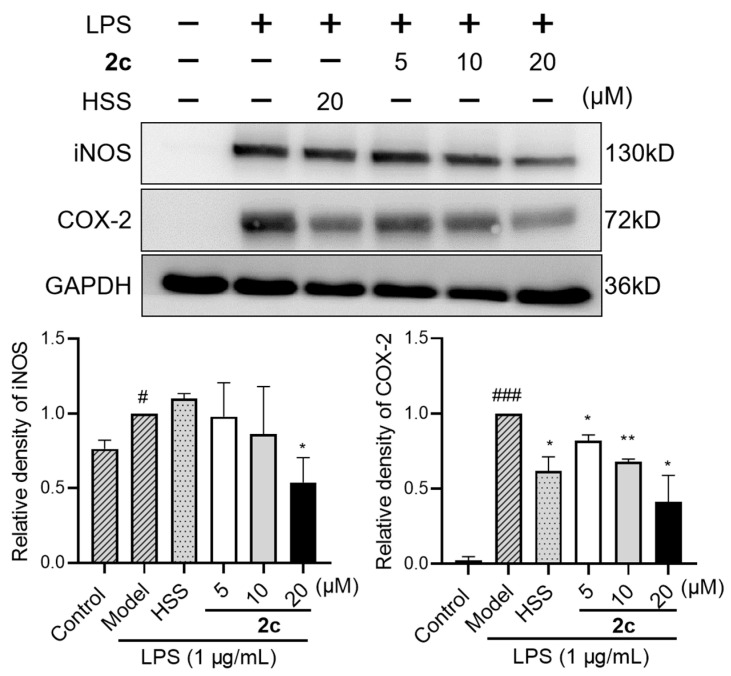
Effects of derivative **2c** on the LPS-triggered generation of iNOS and COX-2 in RAW264.7 macrophages. The macrophages were pre-treated with **2c** (5, 10 and 20 μM) or HSS (20 μM) for 2 h before 24 h of stimulation with LPS (1 μg/mL). Data are expressed as the means ± SD (n = 3): # *p* < 0.05, ### *p* < 0.001 vs. the control group; * *p* < 0.05, ** *p* < 0.01 vs. the model group.

Inflammation-associated enzymes including iNOS and COX-2 are generated and mediated by the activation of NF-κB [45]. Moreover, their abnormal gene overexpression promotes chronic inflammation [46]. Additionally, iNOS is a critical inflammatory mediator that regulates LPS-triggered NO synthesis [39,46]. To confirm the activity of derivative **2c**, we next evaluated its ability to inhibit the LPS-triggered generation of iNOS and COX-2 by performing Western blotting. Compared with the control, treatment with LPS (1 μg/mL) for 24 h rapidly triggered the generation of iNOS and COX-2 in macrophages (Figure 6). Treatment with **2c** significantly inhibited the LPS-triggered generation of iNOS and COX-2 (*p* < 0.05) in a dose-dependent manner (5–20 μM), which was in line with the result of the NO-inhibitory activity of **2c** (Figure 3). Notably, **2c** exhibited better ability in inhibiting LPS-triggered generation of iNOS and COX-2 than did HSS, thereby confirming its anti-inflammatory activity.

### 2.7. Inhibition of LPS-Triggered Activation of NF-κB 

**Figure 7:**
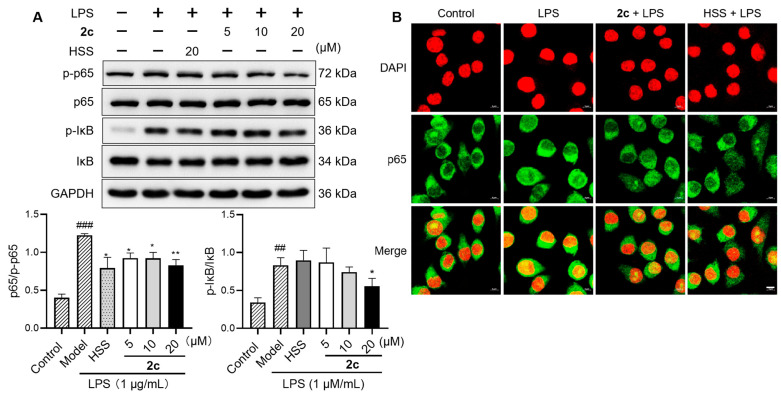
Effects of derivative **2c** on the LPS-triggered activation of NF-κB in RAW264.7 macrophages. The macrophages were pre-treated with **2c** (5, 10, and 20 μM) or HSS (20 μM) for 2 h before 2 h of stimulation with LPS (1 μg/mL). Data are expressed as the means ± SD (n = 3): ## *p* < 0.01, ### *p* < 0.001 vs. the control group; * *p* < 0.05, ** *p* < 0.01 vs. the model group. Scale bar: 5 μm.

Inhibition of the phosphorylation of kappa B (IκB) and p65 is a key marker to initiate the activation of NF-κB [4,47]. Therefore, the effects of **2c** on the phosphorylation of these two were determined. Compared with the control, treatment with LPS (1 μg/mL) for 2 h largely triggered the phosphorylation of IκB and p65 in macrophages (Figure 7A). Derivative **2c** significantly inhibited the LPS-triggered phosphorylation of IκB and p65 (*p* < 0.05) in a dose-dependent manner, and the effect was better than that of HSS. During the activation of NF-κB, phosphorylated p65 undergoes nuclear translocation in the form of the p65–p50 heterodimer, thereby upregulating the expression of genes associated with inflammation [4]. Thus, an immunofluorescence assay was performed to evaluate the effect of **2c** on the LPS-triggered nuclear translocation of the p65–p50 heterodimer. Compared with the control, treatment with LPS (1 μg/mL) for 2 h triggered nuclear translocation (Figure 7B), which was inhibited by derivative **2c**, similar to HSS. These findings indicated that **2c** may exert anti-inflammatory effects by inhibiting the activation of NF-κB.

### 2.8. CETSA

**Figure 8:**
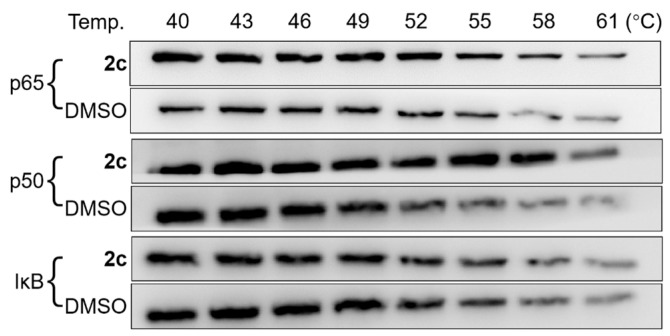
CESTA assessment of derivative **2c** in RAW264.7 macrophages. Immunoblotting results for p65’s and p50’s thermal aggregation curves from samples treated with **2c** (150 μM) or DMSO as a control.

To determine whether **2c** inhibited the activation of NF-κB by directly interacting with NF-κB, a CETSA was performed to analyze the NF-κB–**2c** interactions in cell lysates. Direct interactions between them alter the overall resistance of NF-κB to thermal denaturation, thereby changing the soluble level of NF-κB at a high temperature. The CETSA results are presented in Figure 8. Compared with treatment with a solvent (dimethyl sulfoxide, DMSO), treatment with **2c** stabilized p65 and p50 at high temperatures (≥52 °C). Conversely, no detectable differences were observed between both treatment strategies regarding the stabilization of IκB under the same conditions. These findings indicated that **2c** directly interacts with NF-κB.

### 2.9. Molecular Docking

**Figure 9:**
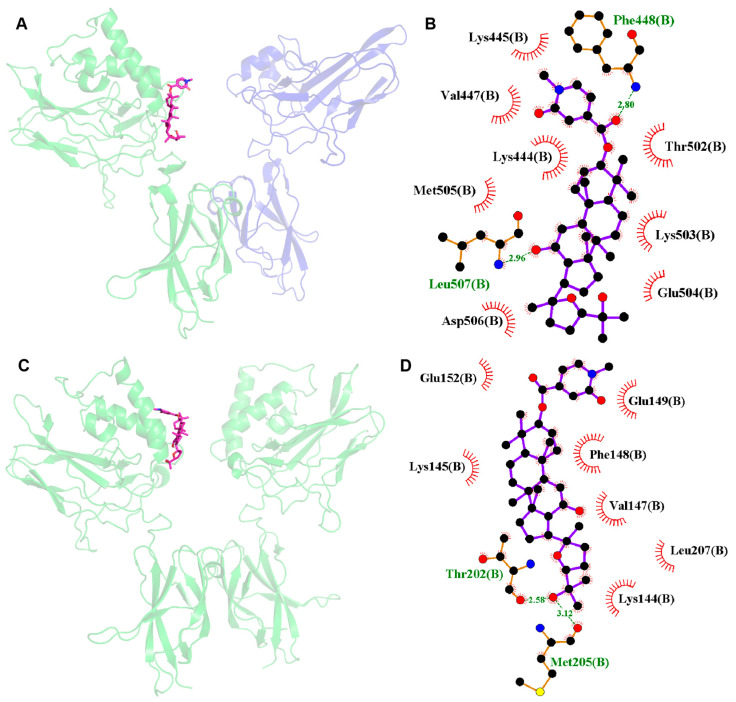
Predicted binding modes of **2c** with (**A**,**B**) the p65–p50 heterodimer (PDB: 1VKX) and (**C**,**D**) the p50 homodimer (PDB: 1NFK). (**A**,**C**) Best docked positions of **2c** in the binding sites of 1VKX and 1NFK. (**B**,**D**) Details of the interactions: hydrophobic interactions and hydrogen bonds are indicated by red eyelashes and green dashed lines.

Molecular docking analysis was performed using the p65–p50 heterodimer (PDB: 1VKX) and p50 homodimer (PDB: 1NFK) to elucidate the molecular mechanism of **2c** in reducing inflammation via NF-κB. The binding sites of **2c** in the two dimers with the highest estimated binding affinity are depicted in Figure 9. Derivative **2c** bound to both dimers at a similar site on the p50 subunit via hydrophobic interactions. Hydrogen bonding was observed in the p50 homodimer between the hydroxy group at C-25 and Thr202 (-OH⋯OC-Thr202, 2.58 Å), in the p65–p50 heterodimer between the hydroxy group at C-12 and Leu507 (-O⋯HN-Leu507, 2.96 Å), and between the ester linker and Phe448 (-CO⋯HN-Phe448, 2.80 Å). In the p50 homodimer, the **2c** binding site exhibited the function of binding to DNA and maintaining the stability of the homodimer [34]; thus, p50 was stabilized by **2c** at a high temperature in the CETSA. The p50 homodimer is an atypical NF-κB species, which exhibits the anti-inflammatory activity by impeding the expression of NF-κB’s target gene [4]. Therefore, the binding of **2c** to the p50 homodimer in order to maintain the homodimers’ stability is one of the potential molecular mechanisms underlying its anti-inflammatory activity. In the p65–p50 heterodimer, the **2c** binding site was close to the acidic tail binding site [33,48] of the p65 subunit and IκB, which probably impeded the phosphorylation of p65, thereby suppressing inflammation (Figure 9A,B). Such interactions enhanced the p65–p50 heterodimer’s stabilization at a high temperature, resulting in the stabilization of p65 in the CETSA. The p65–p50 heterodimer is the active form of NF-κB. The phosphorylation of the p65 subunit of the heterodimer promotes its nuclear translocation and transactivates major proinflammatory genes associated with inflammation. Thus, another potential molecular mechanism of anti-inflammatory activity of **2c** is that it binds to the p65–p50 heterodimer to impede the phosphorylation of p65. However, these molecular mechanisms of **2c** should be further confirmed.

## 3. Materials and Methods

### 3.1. Virtual Screening and Molecular Docking Analysis

The p65–p50 heterodimer (PDB: 1VKX) and the p50 homodimer (PDB: 1NFK) were used as targets for the virtual screening of potential active pyxinol derivatives by molecular docking using AutoDock 4.2.6. The protein structures were established by removing ligands, DNA, water molecules, and other heteroatoms and adding protons and Gasteiger charges. Ligand docking was performed on the active sites of NF-κB. The grid boxes were set at a dimension of 100 × 90 × 90 points with a prod point spacing of 0.508 Å on 1NFK and a dimension of 60 × 70 × 60 points with a prod point spacing of 0.661 Å on 1VKX. The highest estimated binding affinity between the pyxinol derivatives and NF-κB was calculated from 100 replicates per derivative. Predicted binding modes between **2c** and 1NFK or 1VKX were drawn using PyMOL 2.6 and LigPlus 2.2.

### 3.2. Chemistry

All reagents were purchased from commercial suppliers in China and were used as received. Flash column chromatography was performed to purify the synthesized compounds over 200–300 mesh silica gel. The JEOL-ECA400 spectrometer (JEOL, Tokyo, Japan) was used for ^1^H and ^13^C NMR with tetramethylsilane (^1^H: 0.0 p.p.m.) in CDCl_3_ (^13^C: 77.0 p.p.m.) as an internal reference. Scientific-Q Exactive (Thermo Fisher Scientific, Waltham, MA, USA) and SGW-3 were used for HRMS and optical rotation analysis, respectively.

### 3.3. Synthesis of ***2a**–**2h***

Pyxinol (**1**) was prepared and further purified by recrystallization following previously published procedures [16,30,38]. Pyxinol (30 mg, 0.063 mmol) was dissolved in dried DMF or THF (1.0 mL); subsequently, the corresponding carbolic acid (0.094 mmol), EDCI (36 mg, 0.188 mmol), and DMAP (2 mg, 0.016 mmol) were added at 0 °C in the presence of argon. After stirring at room temperature (RT) for a day, the reaction mixture was diluted by adding EtOAc, washed using sat. NaHCO_3_ aq., dried using Na_2_SO_4_, and purified by performing flash column chromatography to yield **2a**–**2h**.

Compound **2a**, white solid, yield: 75%; m.p.: 167–168 °C; ${\left[\alpha \right]}_{D}^{26}$ +28.7 (*c* 1.0, CHCl_3_); ^1^H NMR (400 MHz, CDCl_3_): δ 8.29 (d, J = 9.1 Hz, 2H), 8.19 (d, J = 8.8 Hz, 2H), 4.76 (dd, J = 10.2, 6.0 Hz, 1H), 3.86 (dd, J = 8.9, 6.7 Hz, 1H), 3.54 (td, J = 10.4, 4.7 Hz, 1H), 2.21 (td, J = 10.0, 3.1 Hz, 1H), 2.10–0.94 (m, 21H), 1.28 (s, 3H), 1.28 (s, 3H), 1.10 (s, 3H), 1.02 (s, 3H), 1.01 (s, 3H), 0.94 (s, 3H), and 0.93 (s, 6H); ^13^C NMR (100 MHz, CDCl_3_): δ 164.4, 150.5, 136.4, 130.7 (2C), 123.6 (2C), 86.6, 85.5, 82.9, 71.0, 70.2, 56.2, 52.1, 50.5, 49.5, 48.0, 39.9, 38.7, 38.4, 37.2, 34.8, 32.7, 31.5, 31.3, 28.7, 28.2, 28.0, 27.7, 26.2, 25.1, 23.8, 18.3, 18.3, 16.8, 16.5, and 15.5; HRMS (ESI, positive): *m*/*z* [M+Na]^+^ calculated for C_37_H_55_N_1_O_7_Na^+^ 648.3871, found 648.3872.
Compound **2b**, white solid, yield: 60%; m.p.: 220–222 °C; ${\left[\alpha \right]}_{D}^{24}$ +33.7 (*c* 0.5, CHCl_3_); ^1^H NMR (400 MHz, CDCl_3_): δ 8.81 (d, J = 4.7 Hz, 1H), 8.74 (s, 1H), 8.22 (dd, J = 4.8, 1.2 Hz, 1H), 4.82 (dd, J = 9.1, 7.1 Hz, 1H), 3.86 (dd, J = 8.9, 6.7 Hz, 1H), 3.54 (td, J = 10.5, 4.6 Hz, 1H), 2.21 (td, J = 10.1, 3.1 Hz, 1H), 2.08–0.91 (m, 21H), 1.28 (s, 3H), 1.28 (s, 3H), 1.10 (s, 3H), 1.03 (s, 3H), 1.01 (s, 3H), 0.95 (s, 3H), 0.93 (s, 3H), and 0.92 (s, 3H); ^13^C NMR (100 MHz, CDCl_3_): δ 162.6, 157.4, 149.9, 142.7, 128.4, 117.6, 86.5, 85.4, 84.0, 70.9, 70.1, 56.1, 52.0, 50.4, 49.4, 47.9, 39.8, 38.6, 38.3, 37.1, 34.7, 32.6, 31.4, 31.2, 28.6, 28.2, 27.9, 27.6, 26.1, 25.0, 23.7, 18.2 (2C), 16.7, 16.4, and 15.4; HRMS (ESI, positive): *m/z* [M+Na]^+^ calculated for C_36_H_54_N_2_O_7_Na^+^ 649.3823, found 649.3823.
Compound **2c**, colorless syrup, yield: 80%; ${\left[\alpha \right]}_{D}^{22}$ +37.6 (*c* 1.0, CH_3_OH); 1H NMR (400 MHz, CDCl_3_): δ 7.35 (d, J = 6.9 Hz, 1H), 7.20 (s, 1H), 6.66 (dd, J = 7.0, 1.5 Hz, 1H), 4.66 (dd, J = 10.9, 5.1 Hz, 1H), 3.85 (dd, J = 8.7, 6.7 Hz, 1H), 3.58 (s, 3H), 3.53 (td, J = 10.6, 4.4 Hz, 1H), 2.20 (td, J = 10.0, 4.2 Hz, 1H), 2.08–0.77 (m, 21H), 1.28 (s, 3H), 1.27 (s, 3H), 1.10 (s, 3H), 1.00 (s, 3H), 0.96 (s, 3H), 0.91 (s, 6H), and 0.89 (s, 3H); ^13^C NMR (100 MHz, CDCl_3_): δ 164.2, 162.9, 141.4, 138.5, 122.1, 104.6, 86.5, 85.4, 82.9, 70.9, 70.1, 56.1, 52.0, 50.4, 49.4, 47.9, 39.8, 38.6, 38.2, 37.8, 37.1, 34.7, 32.6, 31.3, 31.2, 28.6, 28.1, 27.9, 27.6, 26.1, 25.0, 23.5, 18.1 (2C), 16.6, 16.4, and 15.4; HRMS (ESI, positive): *m/z* [M+Na]^+^ calculated for C_37_H_57_N_1_O_6_Na^+^ 634.4078, found 634.4062.
Compound **2d**, pale yellow syrup, yield: 62%; ${\left[\alpha \right]}_{D}^{24}$ +28.4 (*c* 0.5, CHCl_3_);^1^H NMR (400 MHz, CDCl_3_): δ 7.67 (dt, J = 8.0, 0.9 Hz, 1H), 7.59 (dd, J = 8.4, 0.7 Hz, 1H), 7.48 (d, J = 0.8 Hz, 1H), 7.44 (ddd, J = 8.5, 7.1, 1.4 Hz,1H), 7.30 (ddd, J = 8.0, 7.1, 0.8 Hz, 1H), 4.78 (dd, J = 9.1, 7.1 Hz, 1H), 4.31 (t, J = 6.6 Hz, 1H), 3.85 (dd, J = 8.8, 6.6 Hz, 1H), 3.54 (td, J = 10.4, 4.7 Hz, 1H), 2.20 (td, J = 10.1, 3.2 Hz, 1H), 2.07–0.88 (m, 21H), 1.28 (s, 3H), 1.27 (s, 3H), 1.10 (s, 3H), 1.01 (s, 3H), 1.01 (s, 3H), 0.94 (s, 6H), and 0.92 (s, 3H); ^13^C NMR (100 MHz, CDCl_3_): δ 159.5, 155.7, 146.0, 128.8, 127.4, 123.7, 122.7, 113.3, 112.4, 86.5, 85.4, 82.1, 70.9, 70.1, 56.1, 52.0, 50.4, 49.4, 48.0, 39.8, 38.6, 38.3, 37.1, 34.8, 32.6, 31.3, 31.2, 28.6, 28.1, 27.9, 27.6, 26.1, 25.0, 23.8, 18.2, 18.1, 16.6, 16.4, and 15.4; HRMS (ESI, positive): *m/z* [M+Na]^+^ calculated for C_39_H_56_O_6_Na^+^ 643.3969, found 643.3953.
Compound **2e**, white solid, yield: 65%; m.p.: 116–117 °C; ${\left[\alpha \right]}_{D}^{24}$ +26.7 (*c* 1.0, CHCl_3_); ^1^H NMR (400 MHz, CDCl_3_): δ 7.53 (dd, J = 9.1, 4.1 Hz, 1H), 7.44 (s, 1H), 7.32 (dd, J = 8.2, 2.5 Hz, 1H), 7.17 (td, J = 9.1, 2.5 Hz, 1H), 4.78 (dd, J = 8.7, 7.6 Hz, 1H), 3.85 (dd, J = 8.8, 6.9 Hz, 1H), 3.54 (td, J = 10.4, 4.4 Hz, 1H), 2.20 (td, J = 10.1, 3.3 Hz, 1H), 2.08–0.85 (m, 21H), 1.28 (s, 3H), 1.27 (s, 3H), 1.10 (s, 3H), 1.01 (s, 6H), 0.93 (s, 6H), and 0.92 (s, 3H); ^13^C NMR (100 MHz, CDCl_3_): δ 159.4 (d, J = 240.8 Hz, 1C), 159.1, 152.0, 147.6, 127.7 (d, J = 11.6 Hz, 1C), 115.7 (d, J = 26.0 Hz, 1C), 113.3 (d, J = 15.4 Hz, 1C), 113.2, 107.7 (d, J = 25.0 Hz, 1C), 86.5, 85.4, 82.4, 70.9, 70.1, 56.1, 52.0, 50.4, 49.4, 48.0, 39.8, 38.6, 38.3, 37.1, 34.7, 32.6, 31.3, 31.2, 28.6, 28.1, 27.9, 27.6, 26.1, 25.0, 23.8, 18.2, 18.1, 16.6, 16.4, and 15.4; HRMS (ESI, positive): *m/z* [M+Na]^+^ calculated for C_39_H_55_F_1_O_6_Na^+^ 661.3875, found 661.3861.
Compound **2f**, white solid, yield: 60%; m.p.: 186–188 °C; ${\left[\alpha \right]}_{D}^{24}$ +30.8 (*c* 1.0, CHCl_3_); ^1^H NMR (400 MHz, CDCl_3_): δ 7.97 (s, 1H), 7.79 (dd, J = 8.9, 4.8 Hz, 1H), 7.53 (dd, J = 9.1, 2.5 Hz, 1H), 7.21 (td, J = 8.9, 2.6 Hz, 1H), 4.71 (dd, J = 10.9, 5.4 Hz, 1H), 3.85 (dd, J = 8.8, 6.6 Hz, 1H), 3.54 (td, J = 10.4, 4.7 Hz, 1H), 2.20 (td, J = 10.1, 3.1 Hz, 1H), 2.10–0.88 (m, 21H), 1.28 (s, 3H), 1.27 (s, 3H), 1.10 (s, 3H), 1.01 (s, 6H), 0.94 (s, 3H), 0.94 (s, 3H), and 0.92 (s, 3H); ^13^C NMR (100 MHz, CDCl_3_): δ 162.2, 160.8 (d, J = 243.7 Hz, 1C), 139.6 (d, J = 9.6 Hz, 1C), 137.6, 136.8, 129.4 (d, J = 3.9 Hz, 1C), 124.0 (d, J = 9.6 Hz, 1C), 116.0 (d, J = 26.0 Hz, 1C), 110.5 (d, J = 22.2 Hz, 1C), 86.5, 85.4, 82.6, 70.9, 70.1, 56.1, 52.0, 50.4, 49.4, 48.0, 39.8, 38.6, 38.3, 37.1, 34.7, 32.6, 31.4, 31.2, 28.6, 28.1, 27.9, 27.6, 26.1, 25.0, 23.7, 18.2, 18.2, 16.6, 16.4, and 15.4; HRMS (ESI, positive): *m/z* [M+Na]^+^ calculated for C_39_H_55_F_1_S_1_O_5_Na^+^ 677.3646, found 677.3632.
Compound **2g**, white solid, yield: 60%; m.p.: 248–249 °C; ${\left[\alpha \right]}_{D}^{24}$ +46.3 (*c* 1.0, CHCl_3_); ^1^H NMR (400 MHz, CDCl_3_): δ 7.97 (dd, J = 6.6, 1.4 Hz, 1H), 7.81 (dd, J = 6.7, 1.5 Hz, 1H), 7.53 (td, J = 7.2, 1.6 Hz, 1H), 7.49 (td, J = 7.2, 1.4 Hz, 1H), 4.74 (dd, J = 11.4, 5.1 Hz, 1H), 3.85 (dd, J = 8.8, 6.9 Hz, 1H), 3.54 (td, J = 10.5, 4.5 Hz, 1H), 2.20 (td, J = 10.1, 3.1 Hz, 1H), 2.10–0.90 (m, 21H), 1.28 (s, 3H), 1.27 (s, 3H), 1.10 (s, 3H), 1.03 (s, 3H), 1.01 (s, 3H), 0.96 (s, 3H), 0.94 (s, 3H), and 0.93 (s, 3H); ^13^C NMR (100 MHz, CDCl_3_): δ 161.2, 138.7, 137.3, 128.1, 126.9, 125.5, 123.9, 122.8 (2C), 86.6, 85.5, 83.1, 71.0, 70.2, 56.2, 52.1, 50.5, 49.5, 48.1, 39.9, 38.7, 38.3, 37.2, 34.9, 32.7, 31.5, 31.3, 28.7, 28.2, 28.0, 27.7, 26.2, 25.1, 23.9, 18.3, 18.3, 16.8, 16.5, and 15.5; HRMS (ESI, positive): *m*/*z* [M+Na]^+^ calculated for C_39_H_55_C_l1_S_1_O_5_Na^+^ 693.3351, found 693.3342.
Compound **2h**, pale yellow syrup, yield: 50%; ${\left[\alpha \right]}_{D}^{24}$ +28.1 (*c* 0.5, CHCl_3_); ^1^H NMR (400 MHz, CDCl_3_): δ 7.24 (d, J = 8.8 Hz, 1H), 7.15 (d, J = 1.9 Hz, 1H), 7.05 (d, J = 2.5 Hz, 1H), 6.85 (dd, J = 8.8, 2.5 Hz, 1H), 4.49 (dd, J = 11.0, 5.2 Hz, 1H), 3.85 (s, 3H), 3.84 (dd, J = 8.8, 6.6 Hz, 1H), 3.73 (d, J = 0.5 Hz, 2H), 3.50 (td, J = 10.6, 4.6 Hz, 1H), 2.18 (td, J = 10.1, 3.1 Hz, 1H), 2.08–0.77 (m, 21H), 1.27 (s, 3H), 1.26 (s, 3H), 1.09 (s, 3H), 0.97 (s, 3H), 0.88 (s, 3H), 0.85 (s, 3H), 0.79 (s, 3H), and 0.75 (s, 3H); ^13^C NMR (100 MHz, CDCl_3_): δ 171.8, 154.1, 131.2, 127.7, 123.6, 112.6, 111.8, 108.7, 100.6, 86.5, 85.4, 81.1, 70.9, 70.1, 56.0, 55.9, 52.0, 50.4, 49.4, 48.0, 39.8, 38.6, 37.9, 37.1, 34.7, 32.6, 31.8, 31.3, 31.2, 28.6, 27.9, 27.9, 27.6, 26.1, 25.0, 23.7, 18.1 (2C), 16.4, 16.4, and 15.4. HRMS (ESI, positive): *m*/*z* [M+Na]^+^ calculated for C_41_H_61_N_1_O_6_Na^+^ 686.4391, found 686.4383.

### 3.4. Cell Culture and NO Release Assay

RAW264.7 macrophages were maintained in the RPMI-1640 complete medium (containing 10% fetal bovine serum and 1% penicillin/streptomycin) and grown in the presence of 5% CO_2_ and 95% O_2_ at 37 °C. The macrophages (5 × 10^4^/well) were seeded in a 96-well plate for 24 h and pre-treated with **2c** or HSS (5, 10, and 20 μM) for 2 h before 24 h of stimulation with LPS (1 μg/mL). The Griess reagent (Beyotime, Shanghai, China) was then added to the culture supernatants, and its absorbance was measured using the SpectraMax-M3 microplate reader (540 nm, Molecular Devices, San Jose, CA, USA) to determine the NO levels.

### 3.5. Cell Viability

The toxicity of the pyxinol derivatives was measured by performing an MTT assay. Cells in the experimental group were prepared in the same way as those prepared for the NO release assay, followed by treatment with an MTT reagent (20 μL, 5 mg/mL) for 4 h. The resulting precipitate was dissolved in DMSO, and its absorbance was measured using the SpectraMax-M3 microplate reader (570 nm).

### 3.6. MitoSOX Assay

Cellular MtROS levels were determined by performing a MitoSOX assay. The macrophages (5 × 10^4^/well) were seeded in a 96-well plate for 4 h and pre-treated with pyxinol derivatives or HSS (5, 10, and 20 μM) for 2 h before 0.5 or 24 h of stimulation with LPS (1 μg/mL). The cells were then washed with phosphate-buffered saline (PBS) and incubated with mitoSOX (5 μM) for 15 min. The cellular fluorescence intensity was measured using the SpectraMax-M3 microplate reader (ex/em: 540/570 nm).

### 3.7. ELISA

The macrophages (5 × 10^4^/well) were seeded in a 96-well plate for 24 h and pre-treated with the pyxinol derivatives or HSS (5, 10, and 20 μM) for 2 h before 6 or 24 h of stimulations with LPS (1 μg/mL). The supernatants of the stimulated samples were used to measure the TNF-α and IL-1β levels using Elabscience ELISA kits (Elabscience, Wuhan, China).

### 3.8. Western Blotting

The levels of the target proteins were determined by performing Western blotting as reported previously [49,50]. Briefly, compound-treated cell lysates were heated with an SDS loading buffer for 10 min at 95 °C and separated by SDS-PAGE. The proteins were transferred and blotted on a polyvinylidene fluoride membrane (Millipore, Billerica, MA, USA) and incubated with primary antibodies obtained from Beyotime (anti-COX-2, anti-iNOS, anti-p-iκB, anti-iκB, anti-p65, anti-p50, or anti-GAPDH) or obtained from Cell Signaling Technology (anti-p-p65, Beverly, MA, USA) and an HRP-conjugated secondary antibody purchased from Beyotime. The proteins were visualized by chemiluminescence according to the Beyotime ECL kit’s protocol and analyzed using ImageJ 1.53k software.

### 3.9. Immunofluorescence Staining

Immunofluorescence staining was performed as reported previously [29]. Briefly, the macrophages were seeded in a glass-bottomed cell culture dish for 24 h and treated with **2c** or HSS (20 μM) for 2 h, followed by 2 h of stimulation with LPS (1 mg/mL). Then the samples were washed with PBS and fixed with 4% paraformaldehyde. After permeabilization with Triton-100 for 10 min, the samples were blocked and incubated with the anti-p65 antibody overnight at 4 °C, followed by incubation with the Beyotime AF488-labeled secondary antibody. After counterstaining with DAPI, the cells were imaged under the LSM-800 confocal microscope (Zeiss, Oberkochen, Germany).

### 3.10. CETSA

The CETSA was performed as reported previously with modifications [35,36]. Briefly, RAW264.7 cells were suspended in cold PBS containing protease inhibitors and lysed by freeze–thaw cycles. The supernatants were collected after centrifugation at 20,000 g and divided into two groups (392.5 μL/group). Each group was treated with **2c** (150 μM) or DMSO and incubated at 37 °C for 0.5 h. Next, each group was divided equally into 8 parts and thermally denaturized at the indicated temperatures (40–61 °C) for 3 min. The denaturized parts were placed at RT for 3 min and frozen in liquid nitrogen. After centrifugation at 12,000× *g* and 4 °C for 20 min, the collected supernatants were examined by performing Western blotting.

### 3.11. Statistical Analyses

Each experiment was performed in triplicate. Student’s *t*-test was performed and *p* < 0.05 was considered statistically significant.

## 4. Conclusions

**Figure 10:**
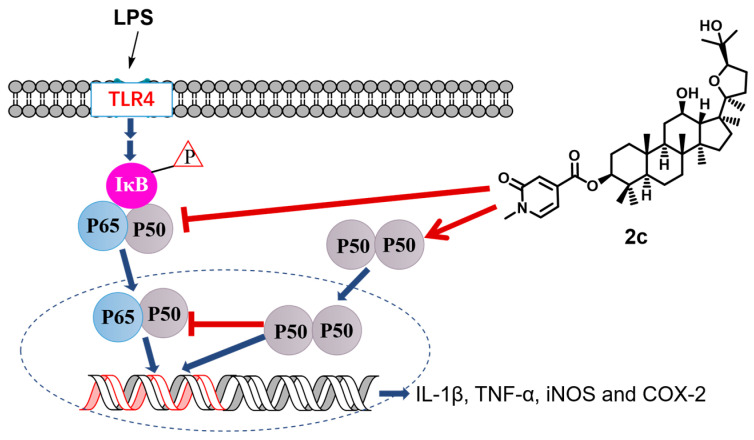
The possible mechanism of **2c** against LPS-triggered inflammation.

Here, we developed pyxinol derivatives targeting NF-κB by performing molecular docking. Their anti-inflammatory activities were examined in LPS-triggered inflammation in macrophages. Derivative **2c**, which exhibited the strongest NO- and MtROS-inhibitory activities with low cytotoxicity, was selected for further study. In LPS-triggered inflammation, **2c** markedly reduced the release of IL-1β, TNF-α, iNOS, and COX-2. Additionally, it suppressed LPS-triggered activation of NF-κB, suggesting that **2c** attenuated inflammation by downregulating abnormal activation of NF-κB. The CETSA confirmed the interactions between **2c** and NF-κB in situ. Molecular docking further revealed that **2c** bound to the p50 subunit in both the p65–p50 heterodimer and the p50 homodimer, thereby stabilizing p50 and p65 at a higher temperature and inhibiting p65’s phosphorylation and p65–p50’s nuclear translocation. In conclusion, these results indicate that **2c** exerts anti-inflammatory effects by directly interacting with NF-κB to downregulate abnormal activation of NF-κB (Figure 10). Further structural optimization of the pyxinol derivatives targeting NF-κB will be performed in the future.

## Figures and Tables

**Table 1:** Docking of selected pyxinol derivatives with the p50 homodimer (PDB: 1NFK) and the p65–p50 heterodimer (PDB: 1VKX).

Compound	Δ*G*_binding_ (kcal/mol)	
	1NFK	1VKX
**2a**	−8.49	−7.71
**2b**	−9.96	−9.38
**2c**	−8.22	−7.98
**2d**	−7.90	−7.44
**2e**	−7.27	−7.49
**2f**	−6.90	−7.42
**2g**	−8.23	−7.56
**2h**	−6.83	−7.53

## Data Availability

Data are contained within the article and Supplementary Materials.

## Supplementary Materials

The following supporting information can be downloaded at https://www.mdpi.com/article/10.3390/molecules29081711/s1, ^1^H NMR and ^13^C NMR spectra of the compounds.
