# Supplementary material for: Synthesis, Anti-Inflammatory Activities, and Molecular Docking Study of Novel Pyxinol Derivatives as Inhibitors of NF-κB Activation
Source: Molecules. 2024 Apr 10;29(8):1711. doi: 10.3390/molecules29081711 (PMC11052049; doi:10.3390/molecules29081711)

Supporting information

Synthesis, Anti-inflammatory Activities, and Molecular Docking Study of Novel Pyxinol Derivatives as Inhibitors of NF-κB Activation

Shuai Tan,[1‡] Zongji Zou,[1‡] Xuwen Luan,[1‡] Cheng Chen,[1] Shuang Li,[1] Zhen Zhang,[1] Mengran Quan,[1] Xiang Li,[1] Wei Zhu,[1,*] and Gangqiang Yang[1,*]

[1] School of Pharmacy, Collaborative Innovation Center of Advanced Drug Delivery System and Biotech Drugs in Universities of Shandong, Key Laboratory of Molecular Pharmacology and Drug Evaluation (Yantai University), Ministry of Education, Yantai University, Yantai, 264005, China

‡ The authors contributed equally.

Corresponding authors：oceanygq@ytu.edu.cn (Gangqiang Yang), zhuwei853@163.com (Wei Zhu)

Table of contents:

^1H and ^{13}C NMR Spectra SI 2

^{1}H and ^{13}C NMR Spectra of **2a**

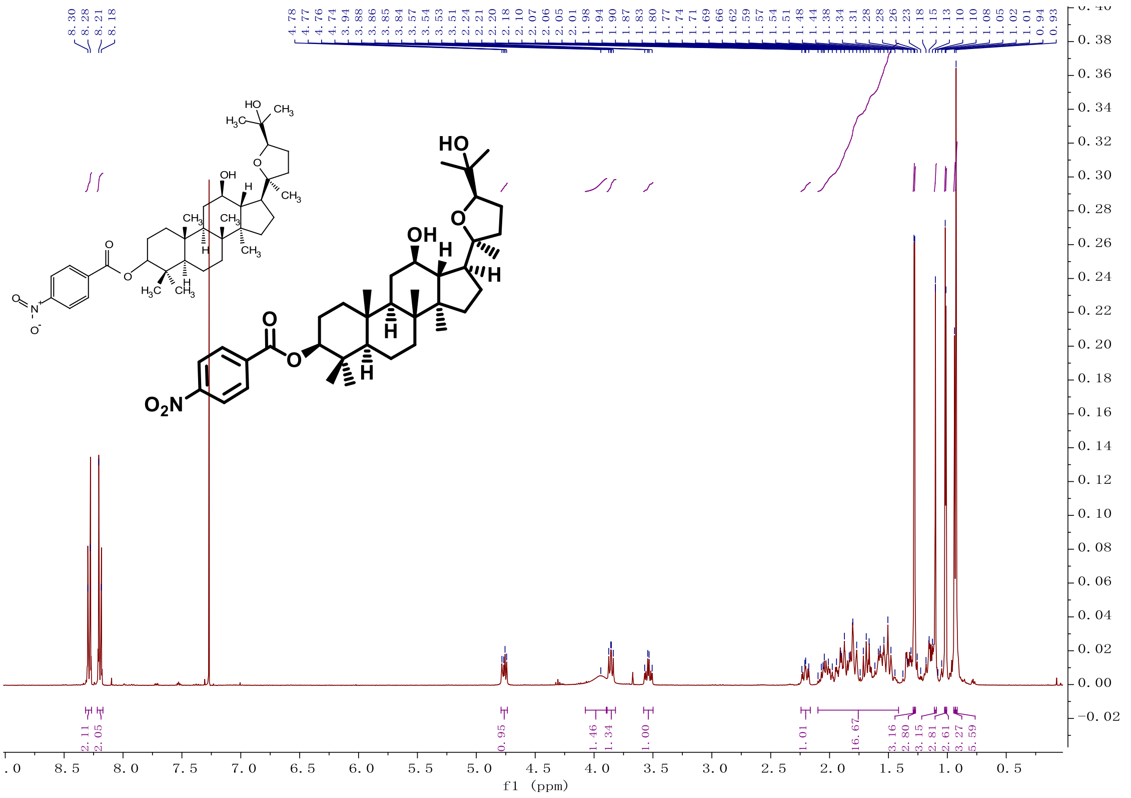

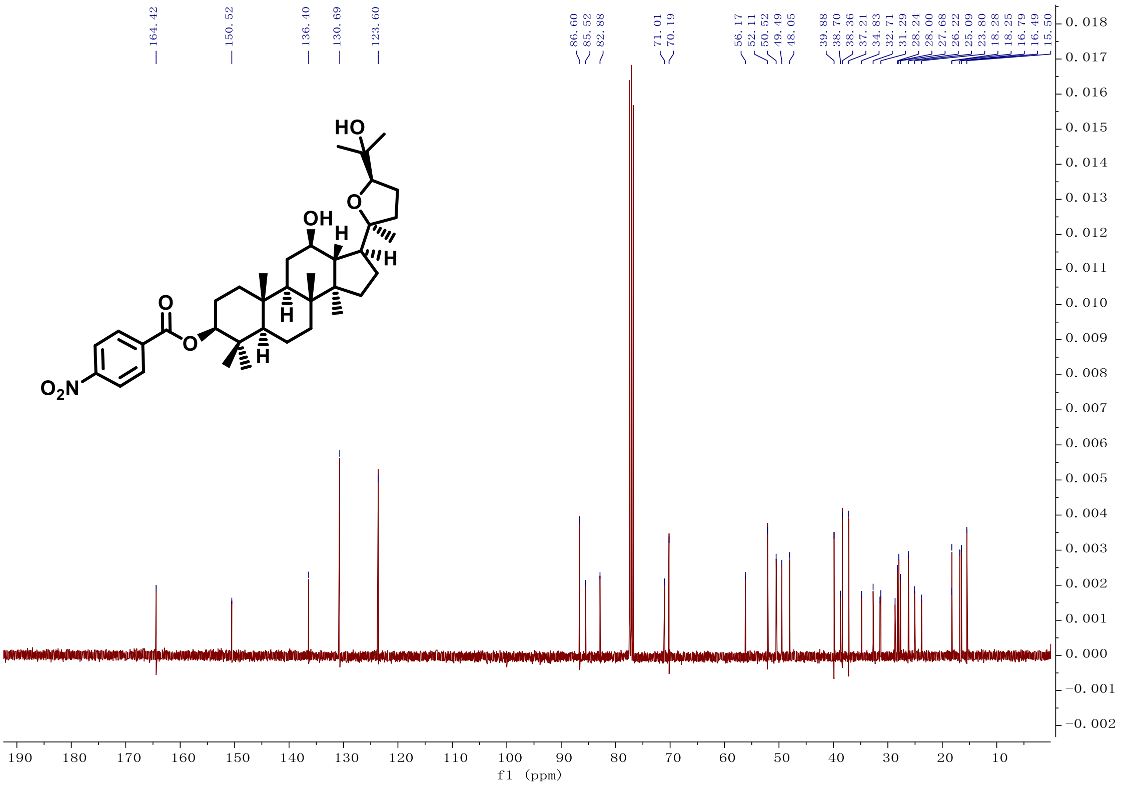

^{1}H and ^{13}C NMR Spectra of **2b**

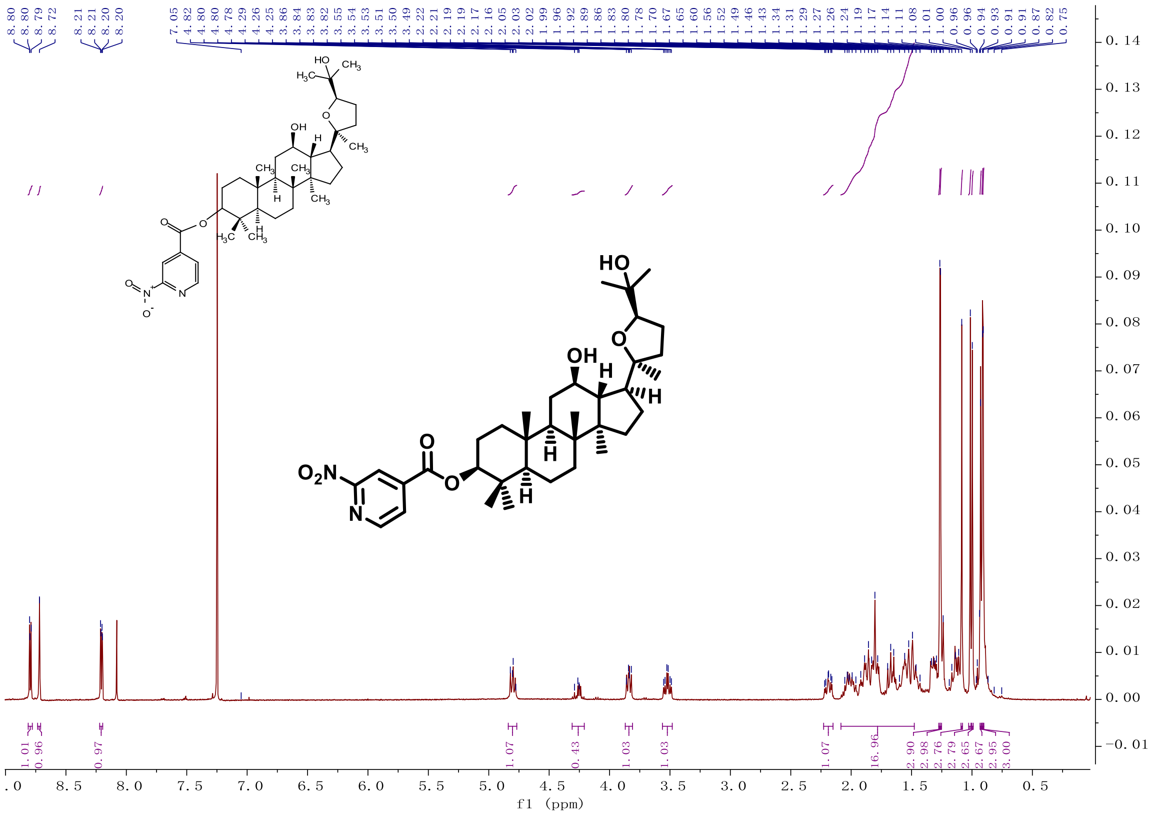

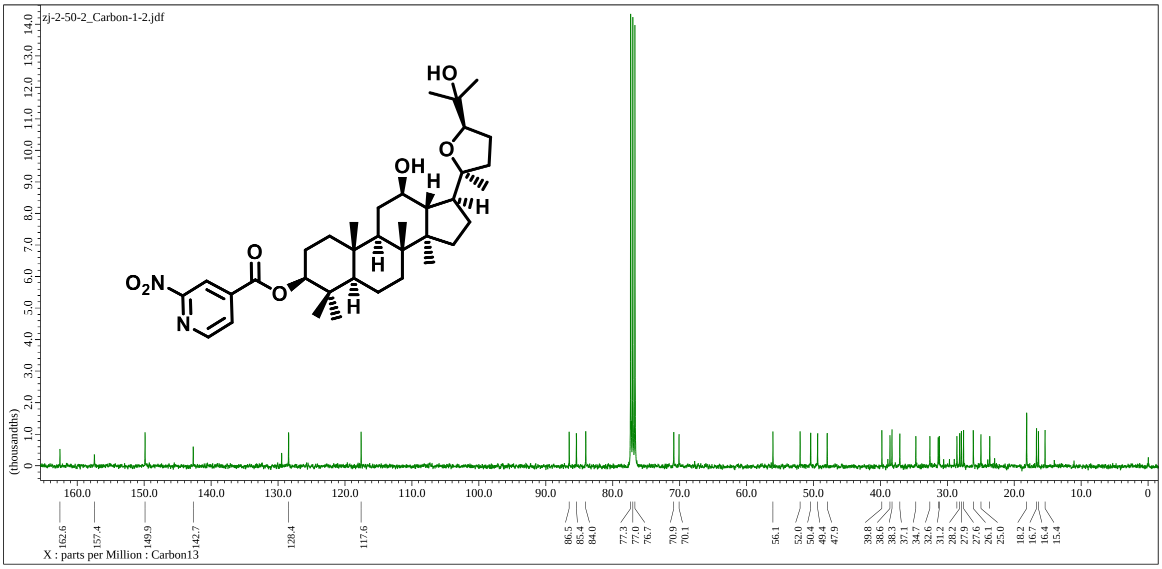

^{1}H and ^{13}C NMR Spectra of **2c**

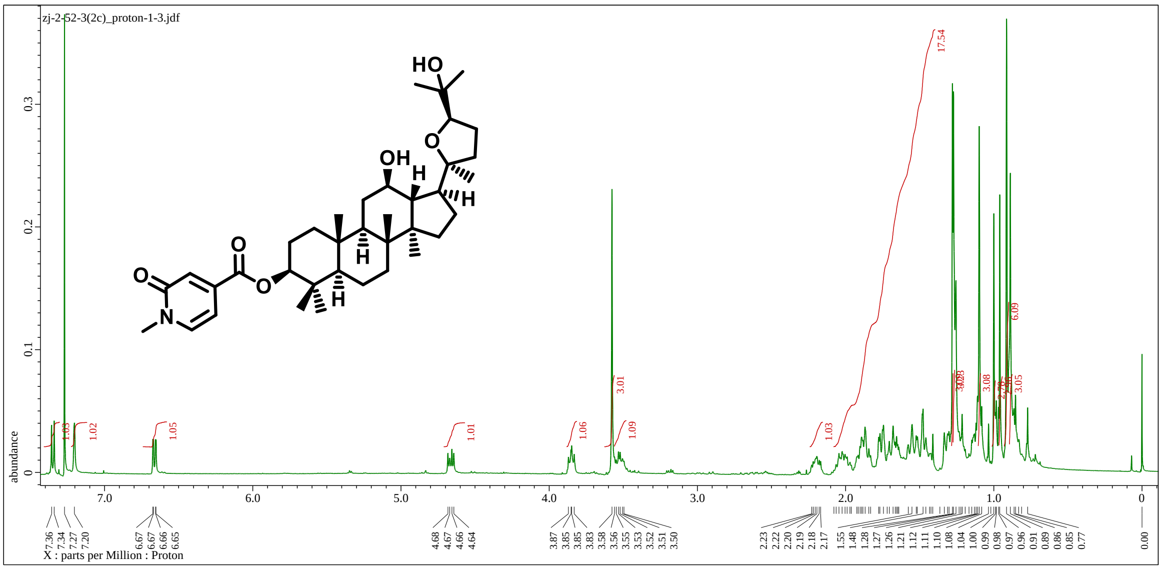

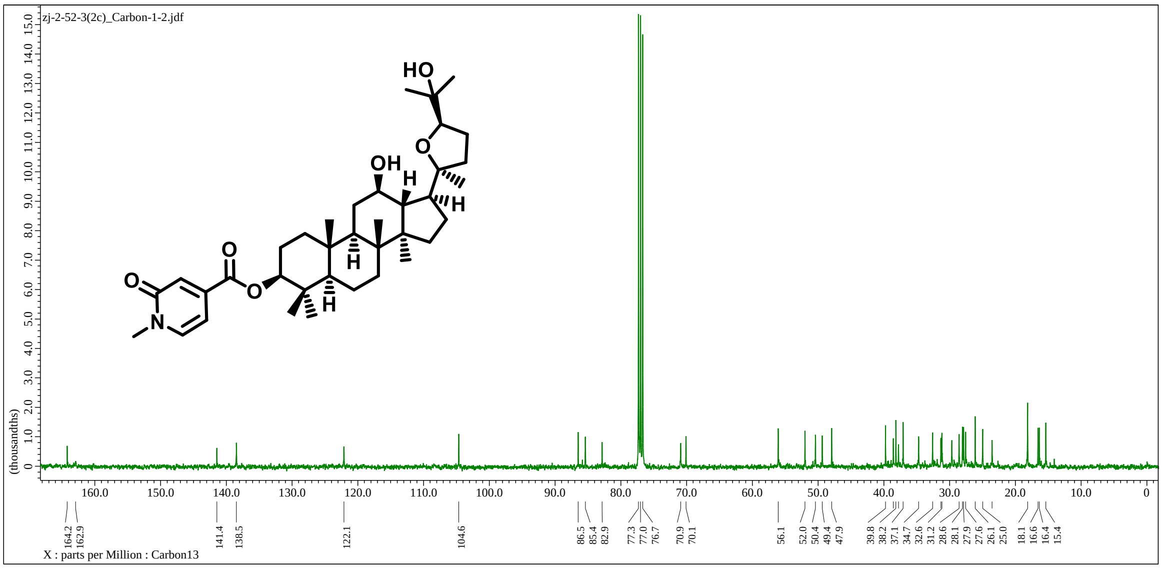

^1H and ^{13}C NMR Spectra of **2d**

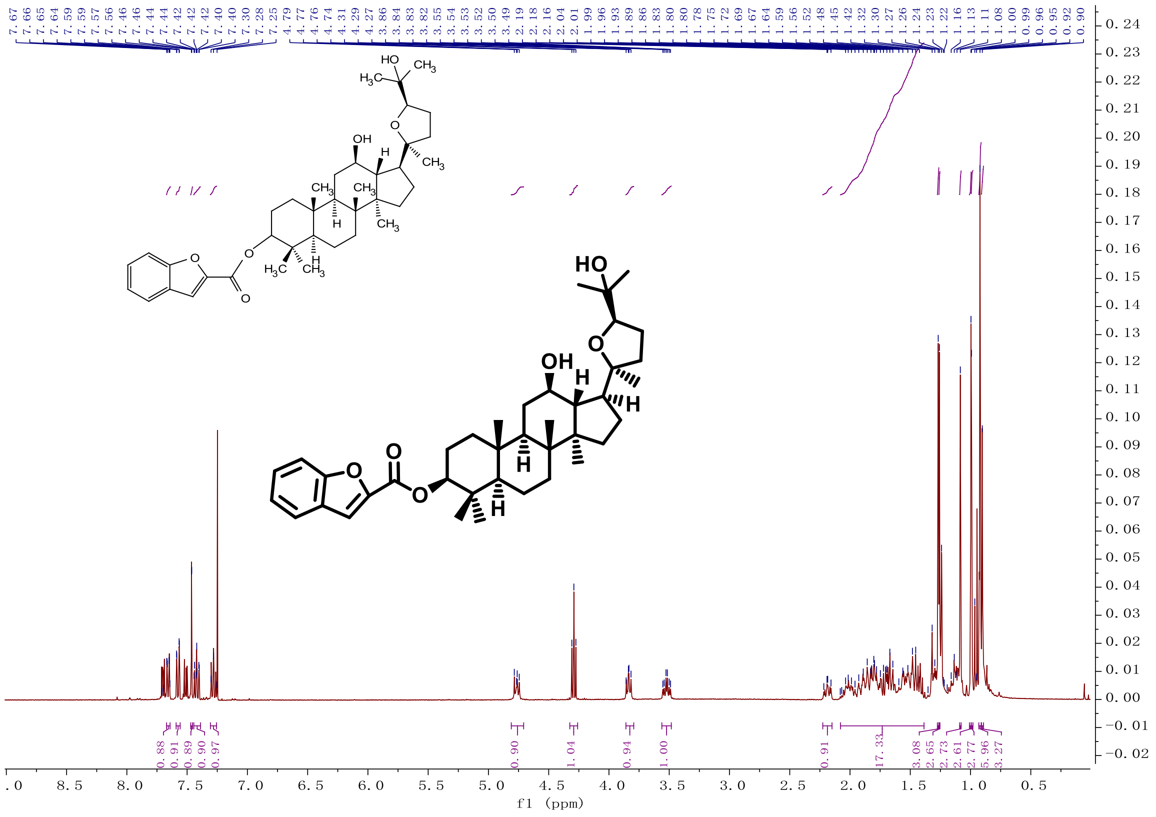

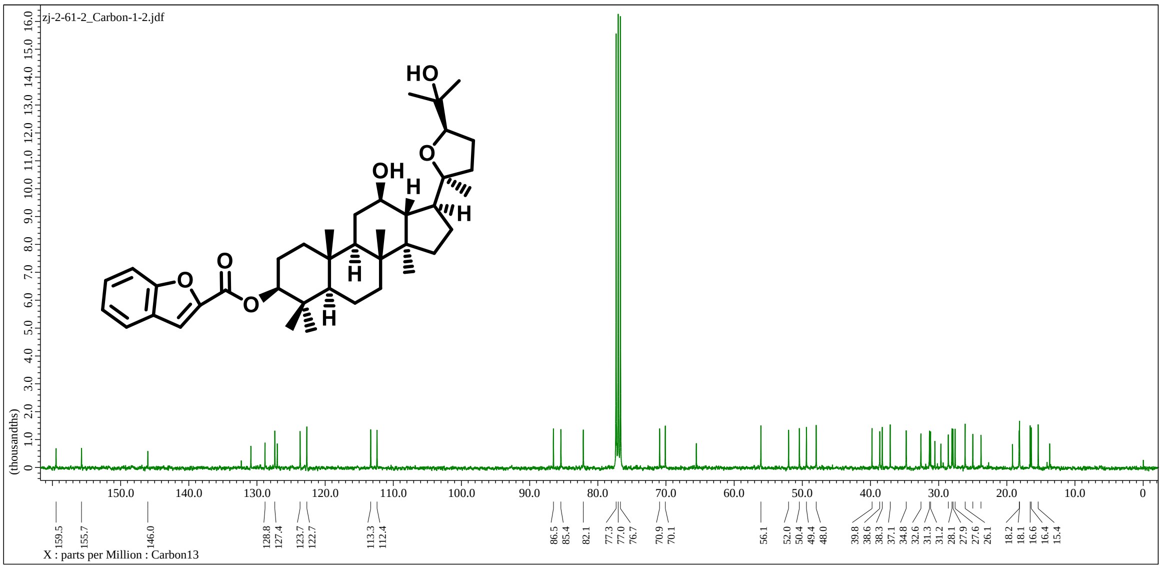

^{1}H and ^{13}C NMR Spectra of **2e**

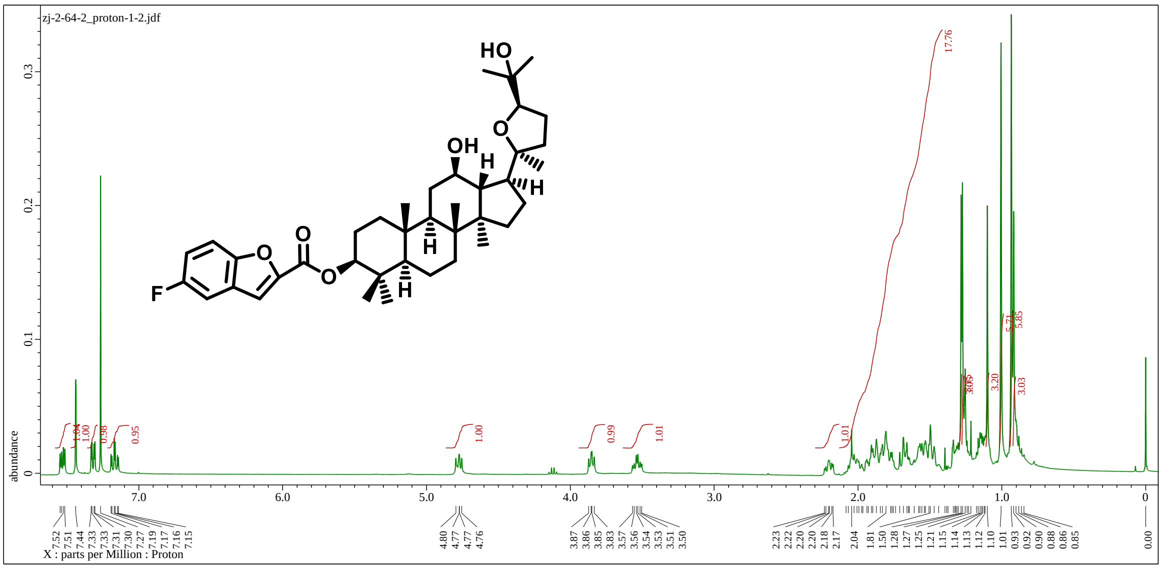

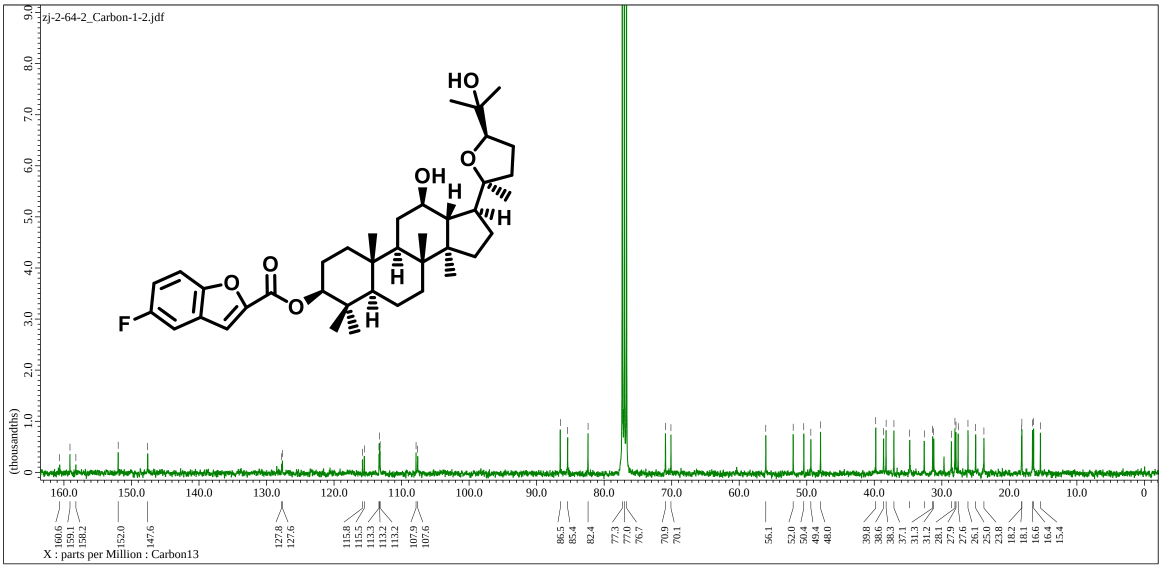

^{1}H and ^{13}C NMR Spectra of **2f**

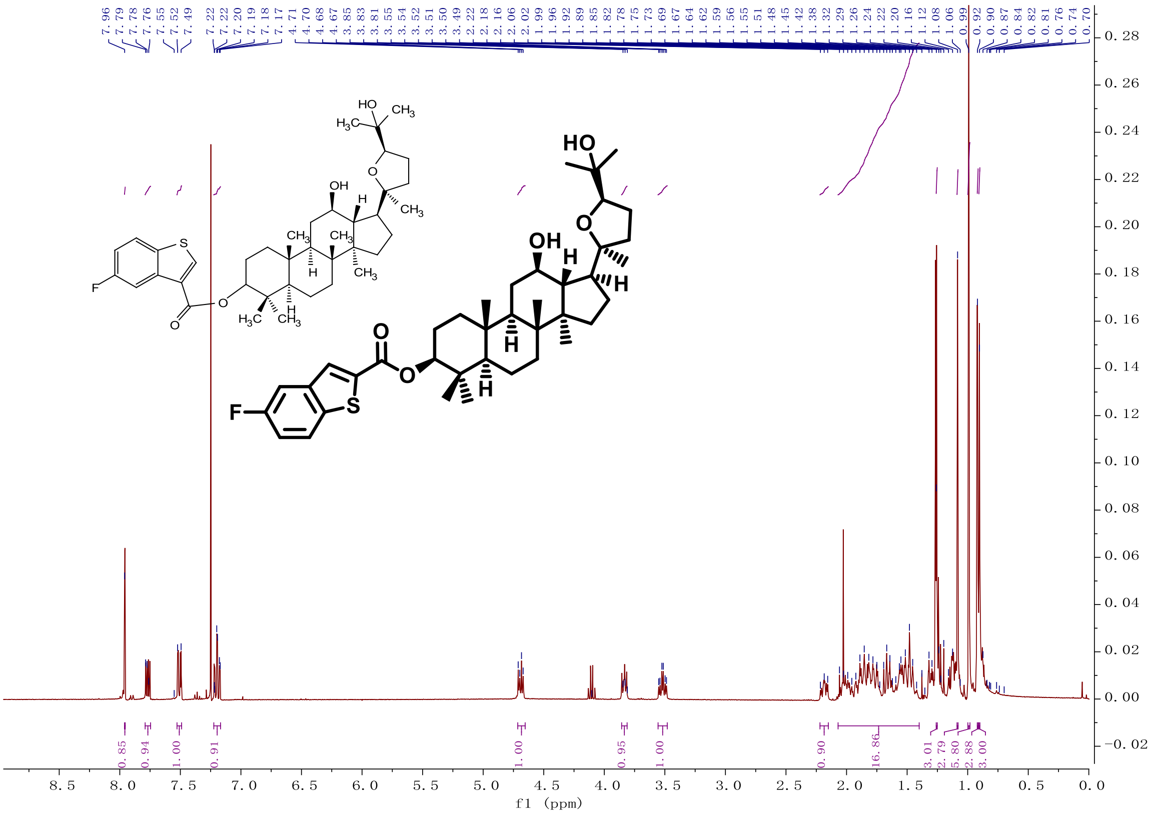

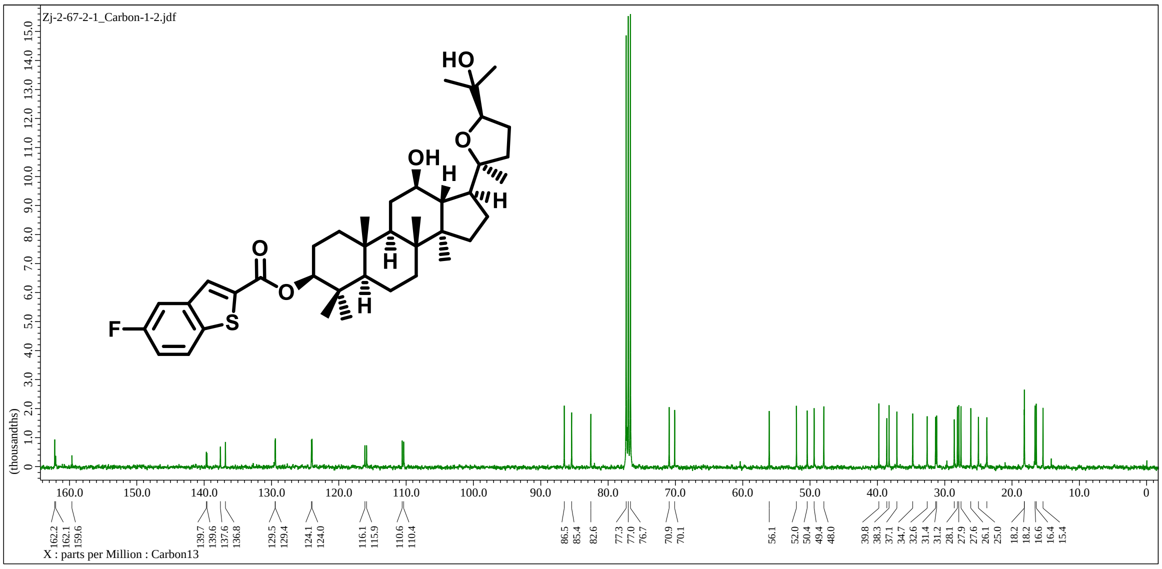

^{1}H and ^{13}C NMR Spectra of **2g**

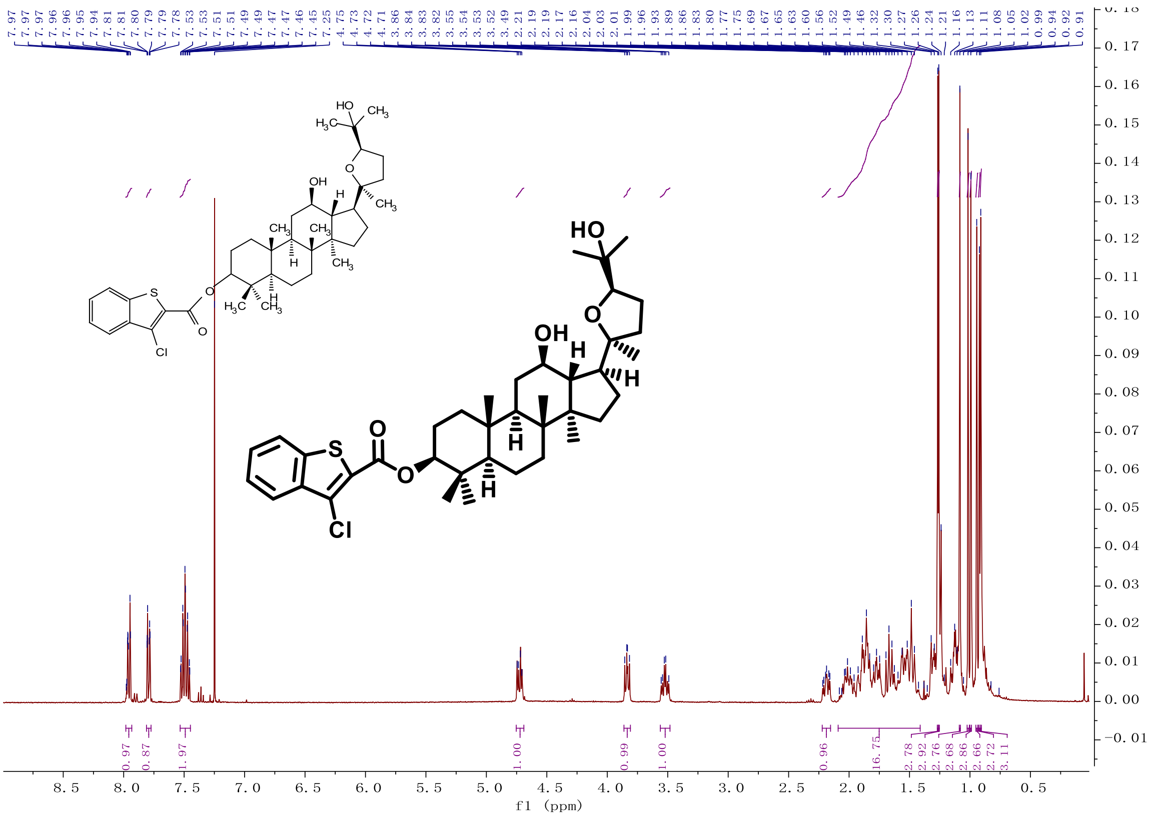

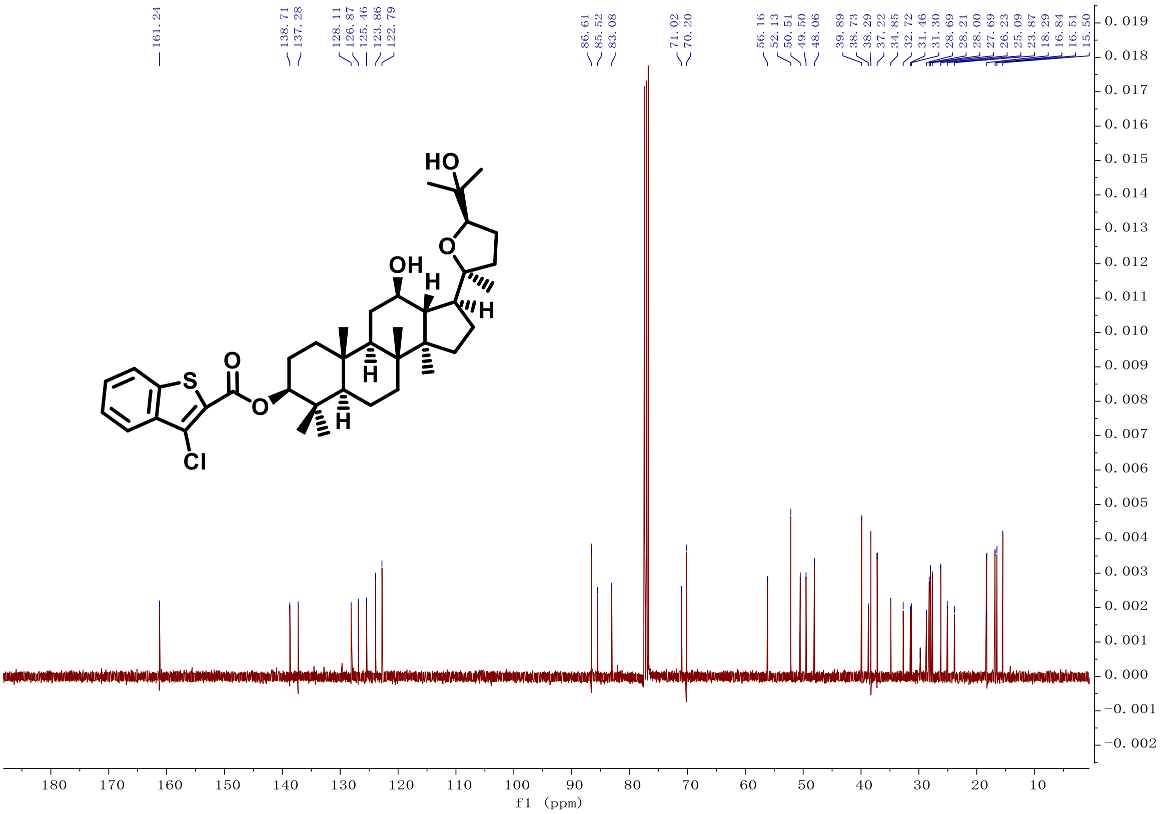

^{1}H and ^{13}C NMR Spectra of **2h**

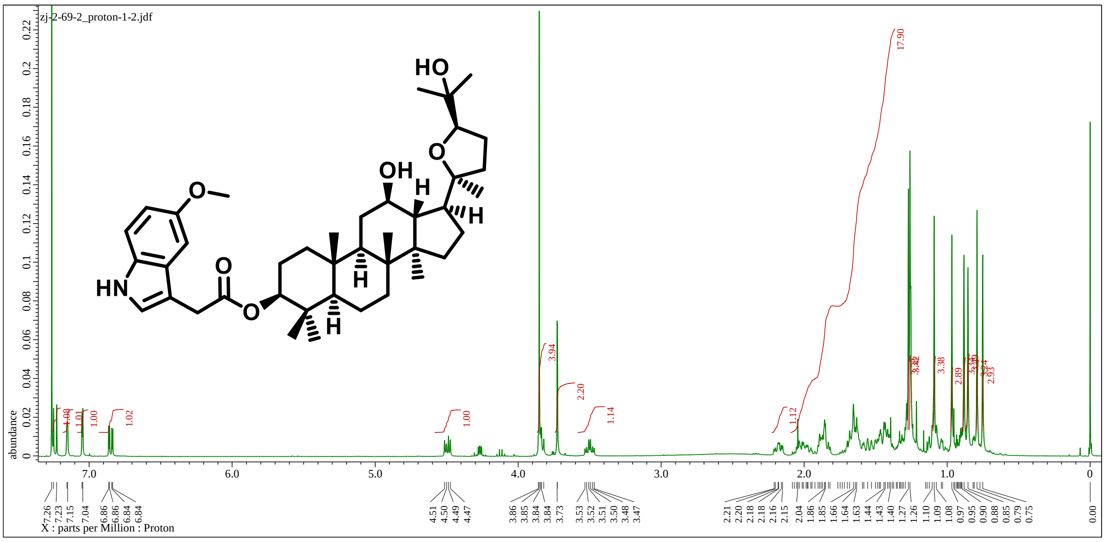

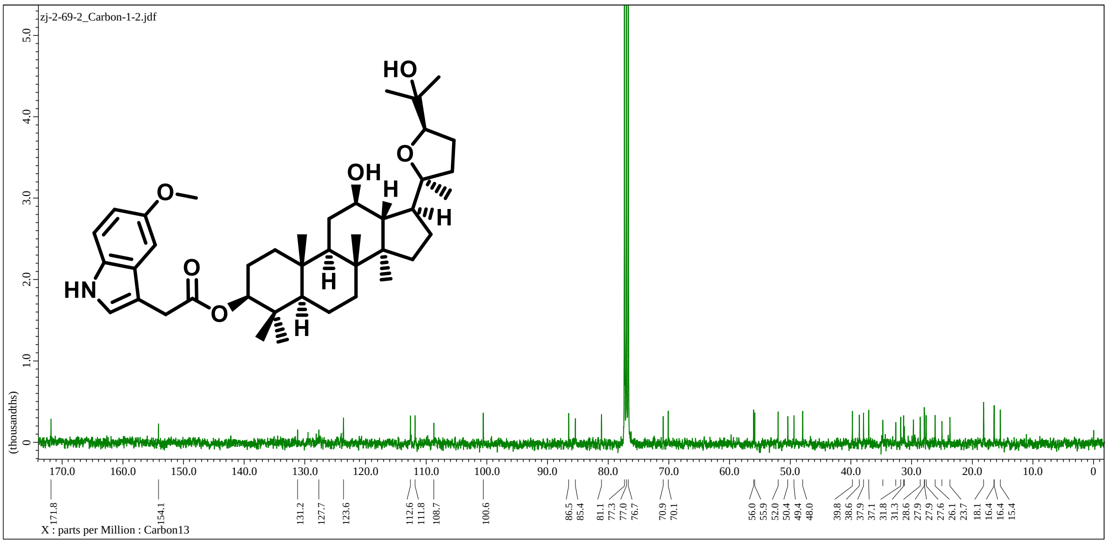

Supplement: Supplementary file 1 [file molecules-29-01711-s001.zip › molecules-2934961-supplementary.pdf]
